# Characterization of the complete chloroplast genome of *Citrus reticulate* (Rutaceae, Citrus)

**DOI:** 10.1080/23802359.2020.1772147

**Published:** 2020-06-04

**Authors:** Ruikai Wu, Tianyi Cao, Jia-Ju Ren, Bao-Jue Liu

**Affiliations:** a Department of Orthopaedics, Zhejiang hospital, Hangzhou, Zhejiang, China;; b Zhejiang Chinese Medical University, Hangzhou, China; c School of Nursing, Beijing University of Chinese Medicine, Beijing, China; d School of Humanities, Beijing University of Chinese Medicine, Beijing, China

**Keywords:** *Citrus reticulata*, *Citrus*, Phylogenetic relationship, Chloroplast genome

## Abstract

The peel of *Citrus reticulata* can be useful as a kind of traditional Chinese medicine in China. The circular complete chloroplast genome of *Citrus reticulata* was 160,101 bp in length with the typical quadripartite structure of angiosperms, including 87,721 bp large single-copy region (LSC), 18,394 bp small single-copy region (SSC) of and a pair of 26,993 bp inverted repeat (IR) regions. The overall nucleotide composition of chloroplast genome sequence is 30.5% A, 31.0% T, C (19.6%), G (18.9%), and the total G + C content of 38.5%. A total of 138 genes were annotated in chloroplast genome of *C. reticulata.* The phylogenetic tree result showed that *C. reticulata* was evolutionarily close to *Citrus sinensis* in the family Rutaceae the genus Citrus by the maximum-likelihood (ML) method.

*C. reticulata* belongs to the family Rutaceae the genus *Citrus* and the peel of *C. reticulata* can be useful as a kind of traditional Chinese medicine that also can be used to treat orthopedic diseases in China. *Citrus reticulata* is one of the most important citrus crops worldwide, which has been one of the centers of mandarin cultivation for four millennia in China (Lun et al. 2018). *Citrus reticulata* as a kind of fruits are attracting increasing attention because of their ease of peeling, nutritional importance, and appetizing flavor. It can be used for medicinal purposes and as healthful food ingredients (Damián-Reyna et al. 2017). In China, the peel of *C. reticulata* can be used as a kind of herb medicine, which can treat orthopedic diseases and enhancing the anti-inflammatory activity and many more diseases (Ho and Lin 2008). Here, the complete chloroplast genome of *C. reticulata* was assembled and characterized, which can use to study the phylogenetic relationship of the genus Citrus plants species, also can provide more traditional Chinese medicine for the future research and utilization.

The fresh samples of *C. reticulata* were obtained from the market near Zhejiang Chinese Medical University located at Hangzhou, Zhejiang and China (119.89E, 30.09 N) and preserved in liquid nitrogen for further research. The total genomic DNA of *C. reticulata* was stored in Zhejiang Chinese Medical University (No. ZJCMU-006). Genomic DNA was extracted from the fresh leaves and sequenced. The quality controlled and removed to the collected raw sequences using FastQC (Andrews 2015). The chloroplast genome of *L. sinense* was assembled using MitoZ (Meng et al. 2019) and annotated using Geneious (Kearse et al. 2012). All the coding and other genes in the chloroplast genome were predicted using CPGAVAS (Liu et al. 2012) and corrected using DOGMA (Wyman et al. 2004).

The circular complete chloroplast genome of *C reticulata* (GenBank Accession number: NK9277641) was 160,101 bp in length with the typical quadripartite structure of angiosperms, including 87,721 bp large single-copy region (LSC), 18,394 bp small single-copy region (SSC) of and a pair of 26,993 bp inverted repeat (IR) regions. The overall nucleotide composition of chloroplast genome sequence is 30.5% A, 31.0% T, C (19.6%), G (18.9%), and the total G + C content of 38.5%. The chloroplast genome of *C. reticulata* contained 138 genes, including 93 protein-coding genes, 37 transfer RNA genes, and eight ribosomal RNA genes. Twenty-two genes were found to contain one IR, including 11 protein-coding genes, seven tRNA genes, and four rRNA genes.

Phylogenetic relationship analysis was carried out using 47 conserved protein-coding genes with those of 14 plant species complete chloroplast genomes that sequences were aligned using MAFFT (Katoh and Standley 2013) and conducted by maximum-likelihood (ML) method using MEGA X (Kumar et al. 2018). 2000 bootstraps values were used in maximum-likelihood (ML) method and at all the nodes under the substitution model. At last, the phylogenetic tree was drawn and edited using MEGA X (Figure 1). The phylogenetic tree result showed that *C. reticulata* was evolutionarily close to *Citrus sinensis* (NC_008334) in the family Rutaceae the genus Citrus. This study will provide more useful information for phylogenetic relationship of the genus Citrus species and also can improve more traditional Chinese medicine for future research and utilization.

**Figure 1.**
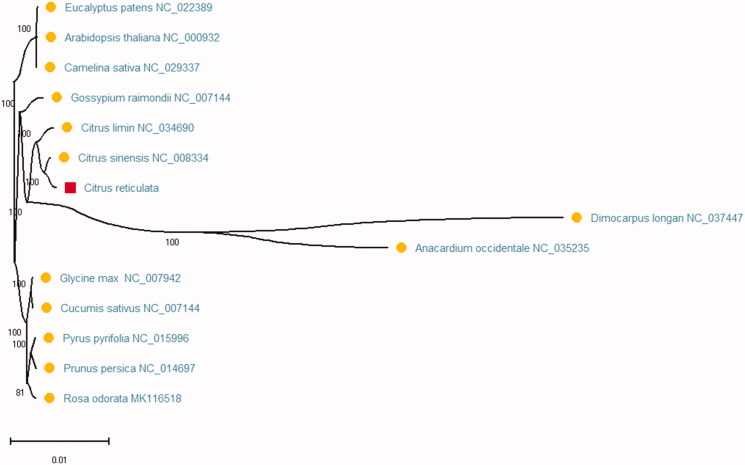
The phylogenetic maximum-likelihood (ML) tree of 14 species based on 47 conserved protein-coding genes. Numbers in the nodes are bootstrap values from 2000 replicates. Accession numbers for tree reconstruction are listed in the figure.

## Data Availability

The data that support the findings of this study are openly available in *Citrus reticulata* at http://doi.org/[doi]. The data that support the findings of this study are available from the corresponding author, upon reasonable request.
